# Supplementary material for: Smartphone overuse, depression & anxiety in medical students during the COVID-19 pandemic
Source: PLoS One. 2022 Aug 30;17(8):e0273575. doi: 10.1371/journal.pone.0273575 (PMC9426930; doi:10.1371/journal.pone.0273575)
Supplement: Attachment — Submitted filename: Response to Reviewers.docx [file pone.0273575.s003.docx]

**Response to the editor’s and reviewer’s comments**

**Journal Requirements:**

When submitting your revision, we need you to address these additional requirements.

1. Please ensure that your manuscript meets PLOS ONE's style requirements, including those for file naming. The PLOS ONE style templates can be found at 
<https://journals.plos.org/plosone/s/file?id=wjVg/PLOSOne_formatting_sample_main_body.pdf> and 
<https://journals.plos.org/plosone/s/file?id=ba62/PLOSOne_formatting_sample_title_authors_affiliations.pdf>.

**Response:** *This manuscript has been revised according to the journal style requirements (please see the revised manuscript).*

2. In your Data Availability statement, you have not specified where the minimal data set underlying the results described in your manuscript can be found. PLOS defines a study's minimal data set as the underlying data used to reach the conclusions drawn in the manuscript and any additional data required to replicate the reported study findings in their entirety. All PLOS journals require that the minimal data set be made fully available. For more information about our data policy, please see <http://journals.plos.org/plosone/s/data-availability>.

Upon re-submitting your revised manuscript, please upload your study’s minimal underlying data set as either Supporting Information files or to a stable, public repository and include the relevant URLs, DOIs, or accession numbers within your revised cover letter. For a list of acceptable repositories, please see <http://journals.plos.org/plosone/s/data-availability#loc-recommended-repositories>. Any potentially identifying patient information must be fully anonymized.

Important: If there are ethical or legal restrictions to sharing your data publicly, please explain these restrictions in detail. Please see our guidelines for more information on what we consider unacceptable restrictions to publicly sharing data: <http://journals.plos.org/plosone/s/data-availability#loc-unacceptable-data-access-restrictions>. Note that it is not acceptable for the authors to be the sole named individuals responsible for ensuring data access.

We will update your Data Availability statement to reflect the information you provide in your cover letter.

**Response:** *The study’s minimal underlying data set was uploaded as a Supporting Information file.*

**Response to Reviewer #1 comments:**

**Abstract**

• Please check for Grammatical errors and ensure the abstract is written in the past tense throughout. For example, the first line could be better phrased to highlight the issue.

**Response:** *We have checked all this sections and provided a better phrasing.*

• In describing the results beware of making claims that students had depression or had addiction. A high score on a questionnaire is only a score, to be diagnosed with depression or addiction will require more than a questionnaire. I suggest you may want to rephrase to ‘high score on the addiction …or a positive relationship ….’.

**Response:** *We have carefully revised the use of these phrases and intended to make a better description of the results.*

• There is too much description of results and this should be more concise with a focus on the relationship between overuse of smartphone and mental disorders

**Response:** *We have rewritten this section focusing on the association of interest.*

• Include an overall summary of what the results mean and why understanding this is important.

**Response:** *We included an overall summary indicating your suggestion.*

• What is missing is an overall statement of the potential contribution of findings.

**Response:** *We have added an overall statement of the potential contribution of findings.*

**Introduction**

Overall this is well-written and puts the study in context with reference to up-to-date and relevant literature, however, strengthening the rationale is likely to improve the manuscript. To this end, you may want to consider further why understanding the association between smartphone use and mental disorders in medical students is important and why this particular group of students. What is unique about medical students and in what is potential value of findings?

**Response:** *We added information regarding the importance of understanding the association between smartphone overuse and mental disorders in medical students, and the potential value of findings, in order to strengthen the rationale of the study.*

It will also be useful to have a clear research question at the end of the introduction and this should be driven by the literature reviewed in the introduction. Given the quantitative nature of the research, a prediction in the shape of an hypothesis should be offered, as you are already indicating a direction in your title. If you wish to be more explorative and not make a prediction than the title may need changing to ‘use’ rather than ‘overuse’.

**Response:** *Following the literature review, we added three research questions at end of the introduction. One of them is the primary question related to the association between smartphone overuse and mental disorders, while the two others are secondary and related to the description of variables of interest. Accordingly, we formulated a hypothesis for each question and offered a predictive form of the main hypothesis.*

What is missing is an overview of research to data on the topic to date. Is this the first study that examined the use of smartphone and mental disorders? In what way your research is similar or different to previous research and what is the research problem or gap it is trying to address?

**Response:** *This is not the first study that examined the association of interest. However, there is no specific information in medical students addressing the problem of smartphone overuse and mental disorders, although literature has reported data in general students and other population. This gap could be necessary to address in the context of the pandemic assuming potential long-term mental disorders in this group. Furthermore, previous research has reported an association between these variables but with no consensus on whether smartphone overuse or mental disorder is the outcome, assuming in some cases a bidirectional association. We add more information to this gap under the assumption that smartphone overuse has a negative effect on mental health.*

**Methods**

***Sample***
• Names of the three schools are included in the manuscript and you may want t indicate whether consent was given by these institutions. Please seek guidance regarding this issue as participating organisations sometimes agree to participate on the assumption that they are not identified in any publications.

**Response:** *Ethical consent was given by only one institution. We decided to deidentify the names of the three schools included in the study to ensure confidentiality of data.*

• I am intrigued by how the exclusion criteria were applied? How did you know if students had depression, received treatment etc? was it self-reported or did you have access to record? It will be useful to have some information on this point.

**Response:** *Depression and whether they received treatment were self-reported. Therefore, we excluded these participants based on their response. We clarified this in the manuscript.*

• There is no information regarding the sample / participants. Were all UG students invited or just from a specific year group? How many from each school took part? Sample characteristics should be provided here.

**Response:** *We added a line explaining that all students were invited to participate in the study. The number of students from each school were clarified.*

***Data collection***

• Providing the educational sessions on excessive use of smartphone is good practice but here you could provide a justification as to why this was done. Was it based on findings? Was it to safeguard participants?

**Response:** *We provided educational sessions to prevent smartphone overuse in all students from the three universities.*

***Variables***

• This section provides information on the outcome, exposure and confounding variables, but it isn’t clear why the variables were defined in such a way as no prediction was made in the introduction regarding direction.

**Response:** *We clarified this methodology by adding more context to the introduction and clarifying the direction of association.*

• You have indicated established reliability of the Smartphone Dependence and addiction Scale. What about validity? Has this been established?

**Response:** *Yes, it has been stablished showing adequate validity. We clarified this part the text.*

• Lines 95-98, you have noted how Insomnia a measured, but what about suicidal ideation, problems related to contagion or loss of a family member, relationship problems and financial problems. Were this assessed via one single question for each, or did you use an established questionnaire?

**Response:** *We used one single question for each of the indicted variables. For insomnia, we used the last question of the PHQ-9. The other variables were measured using a yes/no question.*

• The questions asked are emotive and may trigger a set of emotions, please indicate what measures you put in place and whether support was offered to students

**Response:** *We explained the measures we put in place to safeguard the emotional state of students. Also, we detailed the support we could give participants by providing articles about emotional support, prevention of negative emotional states, and contact numbers for mental health centers.*

***Data analysis***

Once a research question is included in the introduction and a research hypothesis, this will provide justification for the analysis strategy. Please ensure you clearly state which statistical tests were conducted to examine the research hypothesis.

**Response:** *We assured the research question and hypothesis in the introduction. Then, we detailed the reason of each statistical analysis and emphasized which statistical test was conducted to examine the research hypothesis.*

***Ethical consideration***

• Please see comment regarding the names of the participating institutions within the manuscript.

**Response:** *We detailed the name of the institution that gave authorization, but the names of the other institutions were not revealed.*

• In this section you could also mention issues of safeguarding and support offer to students given the emotive nature of study.

**Response:** *We added a paragraph mentioning issued of safeguarding and support offer to students given the emotive nature of study. We also emphasized that participants gave informed consent before continuing with the survey.*

**Results**
Line 114- 115: Information regarding the sample should be moved to the appropriate section in the Methods section.

**Response:** *Information from line 114-115 were moved to the participants section, along with part of their corresponding data from Table 1.*

Was the survey checked for reliability and validity? Cronbach alpha should have been conducted and reported before inferential statistics. Were data normally distributed? Did it meet the assumptions for further regressions?

**Response:** *Our survey had three questionnaires which was not validated in the present study but used previously validated instruments in similar population. Reliability of the instruments in the present study was reported using the Cronbach alpha coefficient (coefficients are presented in Measures subsection). Data regarding depression and anxiety symptoms were not evaluated for normality because we categorized the data for both outcomes as presence or absence (dichotomous response) according to the severity of symptoms. This was in line with the use of Poisson regression models, which omits the normality assumption. The outcomes met the other assumptions for this regression analysis (Y-values are counts, counts are positive integers, counts follow a Poisson distribution, explanatory variables are continuous, dichotomous, or ordinal, and observations are independent).*

Overall this section provides a lot of detail but unfortunately makes for quite a cumbersome read. I suggest it is revised in line with the hypothesis /es that should be included in the introduction.

**Response:** *The results section was revised in line with the hypothesis included in the introduction.*

There are too many long tables, all of which impact the readability of the manuscript so you may want to consider cutting down the information presented ensuring it is focused on the purpose of the study.

**Response:** *Tables were reformatted including only information relevant to the study and focused on the purpose of the study.*

**Discussion**
• Results should not be stated again in the discussion but should be discussed more broadly and in relation to previous literature.

**Response:** *First paragraph regarding overview of results was deleted.*

• In comparing results with previous study, please acknowledge potential differences and similarities that may explain the findings. i.e., comparing to China, Brazil, USA, and pre-pandemic, were the same measures used? Are the studies comparable?

**Response:** *We updated the literature adding information from a meta-analysis and acknowledging limitation for comparison. Also, we detailed the comparison with previous Peruvian studies conducted during the pandemic period (please revise the two paragraphs from lines 269-298).*

• The main issue is wording and you should be careful in stating that participants had depression. As indicated above, a score on a questionnaire is just a score, an indication, it isn’t a diagnosis.

**Response:** *Wording was revised, and we intended to better describe our results.*

• The discussion should provide more of an interpretation of finding and what it means.

**Response:** *We have rewritten the discussion focusing on a more detailed interpretation of findings.*

• A lot of the studies included in the discussion should be summarised in the introduction to indicate research to date in the field.

**Response:** *We summarized studies included in the discussion, as well as added some new information in both sections.*

• Beware of overinterpretation of findings and provide a more concise discussion linked to the research question and hypothesis/es you will include in the introduction.

**Response:** *We carefully revised this section and provided a more concise discussion linked to our main hypotheses.*

• Clearly state in what was=y your study provided some answers to a research problem, in what way it adds to the existing body of literature on the topic, and make suggestions for further research.

**Response:** *We stated how our study provided some answer to a research problem, the way it adds to the existing body of literature, and made suggestions for further research.*

• Please include a discussion of the potential value of findings and how they can inform medical educators. What is the value of findings beyond just telling us that there is a relationship?

**Response:** *We added a discussion of potential value of findings and how they can inform medical educators (lines 320-325).*

**Response to Reviewer #2 comments:**

1. The introduction is poorly written and needs to be completed. In particular, there is too little literature on variable interests (anxiety, depression, insomnia, smartphone use, etc. during the COVID-19 pandemic. Hundreds of articles have been published since 2019, including meta-analyzes and systematic reviews. Authors should rewrite in the introduction, adding In addition, the relationship between depression / anxiety and other variables in the study was described earlier during and earlier during the COVID-19 pandemic, so this information should be presented in the introduction to make direct hypotheses about the expected associations. If the authors are interested in the prevalence rate, information should be provided in previous studies as a basis for hypotheses.

**Response:** *The introduction has been revised and rewritten. In particular, we added more information on the variables of interest during the pandemic, including information from meta-analyzes. We also added a new paragraph describing the association between smartphone overuse and mental disorders (lines 76-92), using relevant studies reported before and during the pandemic. We also used this information to formulate the study hypotheses (lines 104-110).*

2. More information is necessary about the situation of the COVID-19 pandemic in the Peru (e.g., which wave, what the restriction levels, lockdown, e-learning or stationairy classes, etc.) during the data were collected.

**Response:** *More information was added about the situation of the COVID-19 pandemic in Peru (lines 114-118).*

3. How were the inclusion and exclusion criteria controlled? How many students have been excluded because of each criterion?

**Response:** *Selection criteria was controlled by self-report of participants before starting the survey. Students were asked if they met any of the criteria. 38 students were excluded because of age < 18.*

5. How many students refused from the participation in the study? What was the response rate?

**Response:** *Five participants refused to participate in the study. The response rate was 98.6%.*

6. How questionnaires were disseminated?

**Response:** *Questionnaires were disseminated through common WhatsApp groups provided by class presidents from each university.*

7. How were the educational sessions disseminated to the participants (available)? Was the survey anonymous?

**Response:** *Educational sessions were disseminated openly to all students from study universities. This was because we did not have information about the names of the students.*

8. Each questionnaire (PHQ-9, GAD-7, SDAS, ISI) should be comprehensively described, adding information about references to the original and Peruvian version of the instrument, the number of items included in each scale and subscale, response scale shoud be described in details (verbally and digitally), reliablility for each scale and subscale in the original and current sample. Also, demographic variables should be described and all categories of answer should be presented in the Method section.

**Response:** *Each questionnaire was comprehensively described adding information suggested (please revise Measures subsection).*

9. Descriptive analysis should should be showed in the table and commented.

**Response:** *Descriptive analysis was showed in the table and commented (lines 219-225).*

10. The authors stated (lines 103-104): "Student's t test was used after evaluation of normality and homoscedasticity; otherwise, we used the Mann-Whitney U test." but these results are not presented in the manuscript.

**Response:** *We initially used these tests to compare the presence of depressive/anxiety symptoms according to numeric variables such as age and hours of sleep. We have modified this part since these variables did not represent the main findings in our study.*

11. The results of chi-square tests shoud include more statistics (besides p-value), such as Chi-square statistic with df, and effect size (e.g., phi, Cramer's V, or Cohen's d).

**Response:** *We added more detail of the results of chi-square tests, including chi-square statistic, df, and effect size using Cramer's V (Table 3).*

12. It is unclear, how depression and anxiety in Table 2 and Table 3 was assessed (cut-off score should be described in method section, respectively).

**Response:** *A cutoff score for defining the presence of depressive and anxiety symptoms was described in the methods section (lines 191-194).*

13. Discussion should be rewritten, using additional references, which will be added in the Introduction.

**Response:** *We have revised and rewritten the discussion, adding references that are also part of the introduction.*
