# Supplementary material for: Smartphone overuse, depression & anxiety in medical students during the COVID-19 pandemic
Source: PLoS One. 2022 Aug 30;17(8):e0273575. doi: 10.1371/journal.pone.0273575 (PMC9426930; doi:10.1371/journal.pone.0273575)
Supplement: Attachment — Submitted filename: Response to Reviewer 3.docx [file pone.0273575.s004.docx]

**Response to Reviewer #3 comments:**

***Brief message to reviewer 3: We want to thank reviewer 3 for their comments. However, we have noticed that the review of the manuscript was based on the original version and not on the revised one with track changes. Despite of this, we have responded to the comments and added all the suggestions.***

**Introduction**

1. Although the authors have included references from previous reviewers, it is still too brief introduction. The title of the article refers to smartphone overuse and mental disorders. In the introduction, there is a short presentation of both concepts, but it is scarce. It is recommended that the authors include more information and data on the prevalence of smartphone overuse in addition to data on the prevalence of other mental disorders. The introduction talks mainly about depression, so including information on anxiety and insomnia, later evaluated in this study, will provide a greater understanding of the topic.

**Response:** *Thank you for your suggestions. We have previously revised the introduction and focused on depression & anxiety as the outcome measures in this study. We have provided prevalence data on both outcomes as well as on smartphone overuse. We have also highlighted the relationship between depression/anxiety & smartphone overuse, adding current literature on this association. Finally, we stated the research questions and study hypotheses.*

**Methods**

2. Regarding the sample selection criteria, the authors are suggested to be more specific. Are all medical students included, regardless of the course they are taking? Is it an inclusion criterion that they completed the informed consent and responded to the survey? Is access to the smartphone exclusively at home or also in other places?

**Response:** *Thank you. We have specified the information on selection criteria following your suggestions (lines 131-134).*

3. An online survey is used to data collection. This method has the relevant risk of subjectivity, which may bias the data. It is recommended that the author consider this aspect on future research. In addition, it should be included as a limitation of the study.

**Response:** *Thank you. We have included this limitation on lines 337-338.*

4. It is mentioned that following the survey, three educational sessions on the excessive use of smartphones were given. Were these sessions carried out with a specific objective and is the impact of these sessions evaluated in any way?

**Response:** *Thank you. In the latest version of the manuscript, we mentioned the reason for this activity. We have tried to clarify this information further (lines 127-129). Unfortunately, due to logistic limitations we could not gather data on the impact of these sessions.*

5. In this study, different questionnaires are applied to obtain the data. There is a specific questionnaire for depression and another for anxiety. Although other psychopathological variables such as insomnia or suicidal ideation are measured, both the introduction and the results deal mainly with depression and anxiety. Therefore, the authors are asked if the article should not have another title referring specifically to depression and anxiety and not to mental disorders.

**Response:** *Thank you for your feedback. We agree that the title should be more specific according to the two mental health outcomes (depression and anxiety). We have modified the title as suggested.*

**Results**

6. In the results section, reference is made to relationship problems, what do the author mean by this concept? (Page 9, line 135).

**Response:** *Thank you. We have added the definition of this variable to clarify its meaning (lines 181-182).*

**Discussion**

7. The authors are encouraged to review the discussion. They are requested to be uniform when referencing citations in the text. Sometimes the reference is indicated after the punctuation mark, and other times between periods. For example: “However, a study in Korea stated that, rather than addiction, mobile phone users experience “overdependence”, a condition developing in some of them. (21) (Page 11, lines 167-168). And “A unidirectional relationship with depression was also proposed as a possible cause of smartphone addiction. (37, 38). (Page 12, lines 191-193).

**Response:** *Thank you. In the latest version of the manuscript, we have revised all the citations following the journal requirements.*

8. In addition, the authors are suggested to review paragraph 4 of the discussion. The way in which the data are presented is confusing (page 11, lines 169-179).

**Response:** *Thank you. We have previously reviewed this paragraph. Please see paragraphs 2 and 3 of the discussion (pages 15-16, lines 275-304).*
